# *DEK219* and *HSF17* Collaboratively Regulate the Kernel Length in Maize

**DOI:** 10.3390/plants13121592

**Published:** 2024-06-07

**Authors:** Sidi Xie, Ran Tian, Hanmei Liu, Yangping Li, Yufeng Hu, Yubi Huang, Junjie Zhang, Yinghong Liu

**Affiliations:** 1 State Key Laboratory of Crop Gene Exploration and Utilization in Southwest China, Sichuan Agricultural University, Chengdu 611130, China; xiesidichn@163.com (S.X.); tianranchn@163.com (R.T.); liyangping163@163.com (Y.L.); huyufeng@sohu.com (Y.H.); yubihuang@sohu.com (Y.H.); 2 College of Life Science, Sichuan Agricultural University, Ya’an 625014, China; hanmeil@163.com; 3 Maize Research Institute, Sichuan Agricultural University, Chengdu 611130, China

**Keywords:** maize, kernel length, expressing regulation, transcription factor, miRNA

## Abstract

The kernel length is a crucial determinant of maize (*Zea mays* L.) yield; however, only a limited number of genes regulating kernel length have been validated, thus leaving our understanding of the mechanisms governing kernel length incomplete. We previously identified a maize kernel mutant, *defective kernel219* (*dek219*), which encodes the DICER-LIKE1 protein that is essential for miRNA biogenesis. The present study revealed that *dek219* consistently exhibits a stable phenotype characterized by a reduced kernel length. Further analysis indicated that *dek219* may reduce the kernel length by inhibiting the expression of genes involved in regulating kernel length. By employing miRNA-target gene prediction, expression analysis, and correlation analysis, we successfully identified nine transcription factors that potentially participate in the regulation of kernel length under the control of *DEK219*. Among them, the upregulation fold change of *HEAT SHOCK TRANSCRIPTION FACTOR17* (*HSF17*) expression was the highest, and the difference was most significant. The results of transient expression analysis and electrophoretic mobility shift assay (EMSA) indicated that HSF17 can inhibit the expression of *DEFECTIVE ENDOSPERM18* (*DE18*), a gene involved in regulating kernel length. Furthermore, the *hsf17* mutant exhibited a significant increase in kernel length, suggesting that *HSF17* functions as a negative regulator of kernel length. The results of this study provide crucial evidence for further elucidating the molecular regulatory mechanism underlying maize kernel length and also offer valuable genetic resources for breeding high-yielding maize varieties.

## 1. Introduction

Maize (*Zea mays* L.) is one of the most pivotal crops in the world, serving as a vital resource for human nutrition, animal feed, and bioenergy production [1,2]. The exploration of maize kernel size-related genes and the identification of allelic variations of high quality are of paramount importance in enhancing maize yield [3,4,5]. Grain yield, one of the most intricate quantitative traits, is determined by multiple components, including the kernel weight [6]. The kernel weight is significantly influenced by the kernel size, as determined by the length, width, and thickness. A strong positive correlation exists between the grain yield and the kernel size, particularly in terms of kernel length [7,8]. Furthermore, the starch and protein content were also affected by the kernel size [9]. The investigation of the genes and molecular mechanisms associated with kernel length in maize is, therefore, of utmost significance.

Through the use of quantitative trait loci (QTLs) mapping and genome-wide association study (GWAS), numerous QTLs have been identified that are associated with maize kernel length [7,8,10,11,12,13,14,15,16,17,18,19,20]. For instance, Peng et al. successfully identified 11 and 14 QTLs associated with kernel length in two separate F_2:3_ populations, respectively [7]. The study conducted by Li et al. identified a total of 35 QTLs associated with kernel length in 11 recombinant inbred line (RIL) populations [8]. Furthermore, Liu et al. successfully identified 61 QTLs, 55 QTLs, and 138 SNPs for kernel length using the separate linkage mapping (SLM) model, joint linkage mapping (JLM) model, and GWAS model, respectively, through the integration of data from 10 RIL populations [13].

Despite the identification of a substantial number of QTLs associated with kernel length, only a limited number of maize genes influencing this trait have been experimentally validated thus far. For instance, maize *KERNEL LENGTH1* (*KL1*), a gene encoding a single-stranded DNA-binding protein, has been identified as a crucial regulator of kernel length. The overexpression of *KL1* in maize leads to a significant enhancement in kernel length [3]. The gene *KL9*, which encodes the basic leucine zipper60 (bZIP60) transcription factor, also plays a pivotal role in regulating kernel length. It has been reported that the overexpression of *KL9* leads to a significant increase in kernel length, while knockout of this gene results in a substantial decrease in kernel length [4,21]. Furthermore, *DE18* encodes flavin monooxygenase, a pivotal enzyme in the biosynthesis of indole-3-acetic acid (IAA). The mutation of this gene led to a 40% decrease in kernel weight and a significant reduction in kernel length [22,23].

In contrast, numerous genes associated with maize kernel development have been identified through the study of kernel mutants, including *defective kernel 1* (*dek1*), *dek35*, *dek36*, *dek38*, *dek48*, *dek407*, *embryo defective 14*, *pentatricopeptide repeat 6* (*PPR6*), *PPR278*, *opaque11*, *small kernel1* (*smk1*), *smk7a*, and *U6 biogenesis-like1* [24,25,26,27,28,29,30,31,32,33,34,35,36]. These mutants typically exhibit severe phenotypic abnormalities in kernels, such as empty pericarp, embryo lethality, or endosperm abnormality. However, natural variations in these genes may be associated with the maize kernel length. The identification of *Unhealthy Ribosome Biogenesis 2* (*Urb2*), a gene involved in ribosome biogenesis essential for kernel development, was facilitated through the utilization of a maize kernel mutant. Furthermore, significant associations between natural variations in *Urb2* and the kernel length were observed [37].

We previously identified a maize kernel mutant, *dek219*, exhibiting a significant reduction in kernel length. *DEK219* encodes the DICER-LIKE1 protein, a crucial enzyme involved in the biogenesis of miRNAs. Furthermore, candidate gene association analysis showed that natural variations in *DEK219* were significantly associated with the kernel length. In this study, we further identified the genes associated with kernel length downstream of *DEK219* and investigated the molecular mechanism by which *DEK219* regulates kernel length. The findings of this study will contribute to the elucidation of the regulatory mechanism underlying maize kernel length and offer gene resources for the breeding of high-yield maize varieties.

## 2. Results

### 2.1. The dek219 Mutant Exhibited a Significant Reduction in Kernel Length

**Figure 1:**
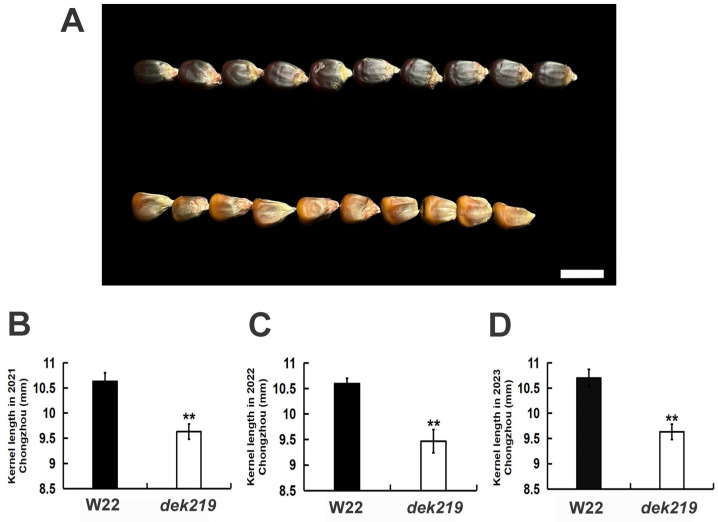
The kernel length of *dek219* has significantly reduction. (**A**) Randomly selected mature W22 (top) and *dek219* (bottom) kernels. Scale bar, 1 cm. (**B**) Kernel length of W22 and *dek219* mature kernels in 2021 Chongzhou. (**C**) Kernel length of W22 and *dek219* mature kernels in 2022 Chongzhou. (**D**) Kernel length of W22 and *dek219* mature kernels in 2023 Chongzhou. ** Significant at *p* < 0.01 by the Student’s *t* test.

The kernel length of the *dek219* homozygous mutant exhibited a significant reduction compared to the wildtype W22. The kernel length of the *dek219* homozygous mutant was measured as 9.63, 9.46, and 9.63 mm in Chongzhou during the years 2021, 2022, and 2023, respectively. The corresponding kernel lengths of W22 were recorded as 10.63, 10.6, and 10.7 mm for the same time period (Figure 1). This indicated that *DEK219* plays a crucial role in the regulation of maize kernel length.

### 2.2. Identification of Pivotal Genes Governing Kernel Length

**Figure 2:**
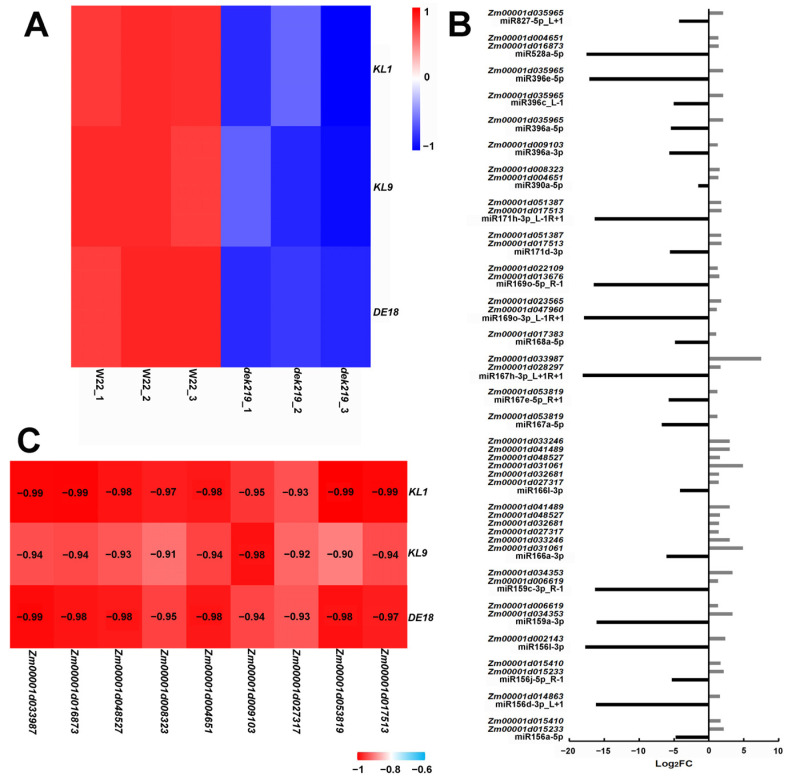
Identification of genes regulating kernel length. (**A**) Heat map depicting the expression levels of *KL1*, *KL9*, and *DE18* in W22 and *dek219*, with 3 biological replicates for each material. These three genes all regulate kernel length, and their expression levels are significantly downregulated in *dek219*. (**B**) Differential expression of miRNAs and their target genes in WT and *dek219*. FC, fold change of the expression of miRNAs/target genes in *dek219* relative to that in WT based on the sequencing data. Black bars represent expression of miRNAs; gray bars represent expression of target genes. (**C**) The correlation between nine transcription factors regulated by miRNA and *KL1*, *KL9*, and *DE18*.

The expression levels of the crucial genes *KL1*, *KL9*, and *DE18* implicated in the regulation of maize kernel length exhibited a significant decrease in *dek219* (Figure 2A). The findings suggested that the reduction in *dek219* kernel length could potentially be achieved through the suppression of gene expression involved in the regulation of kernel length. *DEK219* encodes DICER-LIKE1, a pivotal enzyme in miRNA biogenesis, thereby potentially regulating the expression of genes associated with kernel length through miRNAs. The expression levels of the majority of miRNAs in *dek219* were found to be suppressed [5], implying that miRNAs may not exert direct regulation on genes associated with kernel length but rather modulate their expression through miRNA-transcription factor modules. Transcriptome analysis revealed that the expressions of 155 transcription factors were significantly upregulated in *dek219* (Table S1). Using the miRNA-target gene prediction tool psRNATarget (https://www.zhaolab.org/psRNATarget/, accessed on 1 March 2022, expectation ≤ 4), we found that 27 of the 155 upregulated transcription factors may be the target genes of miRNAs (Table S2). The expression levels of miRNAs potentially regulating these 27 transcription factors are significantly downregulated in *dek219*, exhibiting an inverse correlation with the expression patterns of their target genes (Figure 2B). The correlation between the 27 transcription factors and kernel length genes *KL1*, *KL9*, and *DE18* was further analyzed, revealing a significant negative association with all three kernel length genes (Table S3). Among them, the correlation coefficients between the nine transcription factors and the three kernel length genes were all below −0.90 (Figure 2C). These nine transcription factors may exhibit a closer association with the kernel length genes *KL1*, *KL9*, and *DE18*. Among the nine transcription factors, *HSF17* (*Zm00001d033987*) exhibited the most significant upregulation (181-fold increase) and demonstrated the lowest *p*-value (*p*-value = 2.08 × 10^−101^; Table S1). Therefore, we identified *HSF17* as one of the candidate transcription factors implicated in the regulation of the kernel length genes *KL1*, *KL9*, and *DE18*.

### 2.3. The Expression of DE18 Was Suppressed by HSF17

**Figure 3:**
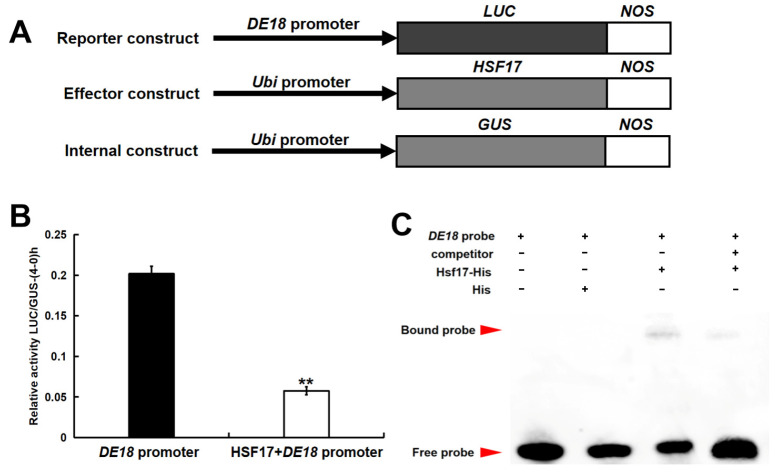
The expression of *DE18* is inhibited by *HSF17*. (**A**) Diagram of the effector, reporter, and internal constructs in transient expression analysis. (**B**) The transient expression analysis has demonstrated that HSF17 exerts a significant inhibitory effect on the activity of the *DE18* promoter. ** Significant at *p* < 0.01 by the Student’s *t* test. (**C**) The electrophoretic mobility shift assay confirmed the specific binding of HSF17 to the ACAAGTTTCT motif.

Our previous study revealed the inhibitory effect of miR167h-3p_L+1R+1 on the expression of *HSF17* [5]. To verify whether the kernel length gene *DE18* is regulated by *HSF17*, transient expression analysis of the *DE18* promoter and *HSF17* was performed. The findings demonstrated that HSF17 effectively suppressed the activity of the *DE18* promoter (Figure 3B). We analyzed the promoters of *DE18* using the plant promoter analysis website Plantpan4.0 (http://plantpan.itps.ncku.edu.tw/plantpan4/index.html, accessed on 16 March 2022). A possible binding motif of the HSF transcription factor, ACAAGTTTCT, was found in the *DE18* promoter. EMSA further verified that HSF17 can bind to the ACAAGTTTCT motif (Figure 3C). The findings demonstrated that HSF17 exerts a direct inhibitory effect on the expression of *DE18* through its binding to the *DE18* promoter.

### 2.4. The Mutant hsf17 Exhibited a Significant Increase in Kernel Length

**Figure 4:**
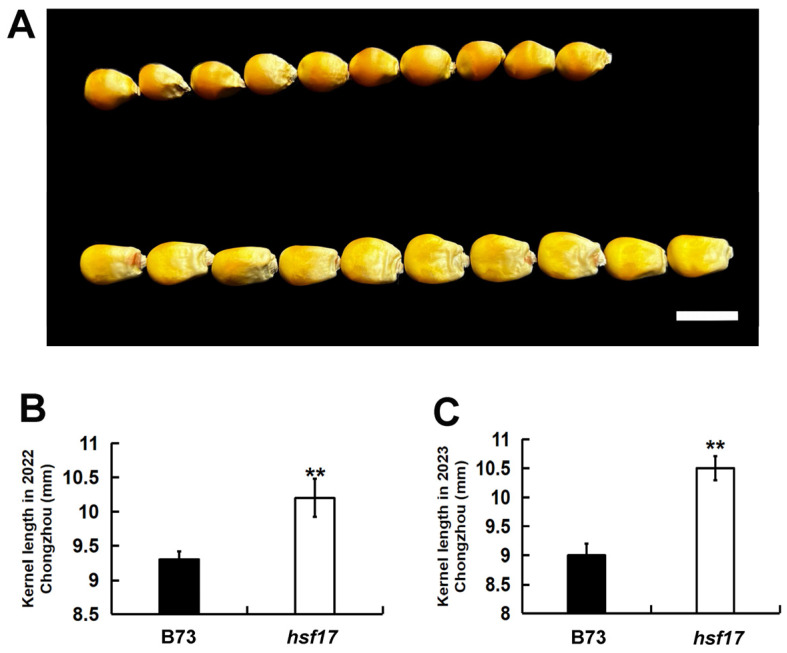
The kernel length of the mutant *hsf17* is significantly increased. (**A**) Randomly selected mature B73 (top) and *hsf17* (bottom) kernels. Scale bar, 1 cm. (**B**) Kernel length of B73 and *hsf17* mature kernels in 2022 Chongzhou. (**C**) Kernel length of B73 and *hsf17* mature kernels in 2023 Chongzhou. ** Significant at *p* < 0.01 by the Student’s *t* test.

We further identified the mutant of *HSF17*. The mutation of *HSF17* involves a G-to-A substitution in exon 3, resulting in the conversion of a tryptophan residue to a premature stop codon, potentially impacting its functionality. The *hsf17* homozygous mutants exhibited a significant increase in kernel length compared to the wildtype B73 (Figure 4). The kernel length of the *hsf17* homozygous mutant was measured as 10.20 and 10.50 mm in Chongzhou during the years 2022 and 2023, respectively. Meanwhile, B73 exhibited corresponding kernel lengths of 9.30 mm and 9.00 mm for the same time period (Figure 4B,C). The findings suggested that *HSF17* functions as a negative regulator of maize kernel length and plays a crucial role in the regulation of this trait.

## 3. Discussion

### 3.1. DEK219 and HSF17 Play a Pivotal Role in the Regulation of Kernel Length

Despite the large number of QTLs for kernel length that have been identified [7,8,11,12,13,14,15,16], thus far, only a few genes that influence kernel length have been validated in maize. The genes *KL1*, *KL9*, and *DE18* have been identified as positive regulators of kernel length, with *KL1* encoding a single-stranded DNA binding protein, *KL9* encoding the bZIP60 transcription factor, and *DE18* encoding flavin monooxygenase [3,4,21,22,23]. The expression levels of these three genes were significantly downregulated in *dek219*, indicating that *DEK219* may regulate the kernel length through a modulation of the expression of kernel length-related genes. Furthermore, based on candidate gene association analysis, *GRAIN SIZE3* (*GS3*), *GS5*, *GRAIN WIDTH AND WEIGHT2* (*GW2*), and CELL WALL INVERTASE1 (*INCW1*) have been identified as being significantly associated with kernel length [13,38,39,40]. The mutant *dek219* exhibits a significant reduction in kernel length, indicating the pivotal role of *DEK219* in the regulation of kernel length. *DEK219* encodes the DICER-LIKE1 protein, which is essential in miRNA biogenesis [5]. The results suggested that the regulatory effect of *DEK219* on kernel length is mediated through the modulation of miRNA expression. We identified nine transcription factors that are under the regulation of miRNAs and are more likely to govern kernel length. Among them, it has been experimentally validated that miR167h-3p_L+1R+1 targets *HSF17* [5]. The mutant of *HSF17* exhibits a significant increase in kernel length. This may be attributed to the negative regulatory role of *HSF17* in gene expression related to kernel length, such as *DE18*. To our knowledge, there have been no previous reports on the involvement of miR167h and *HSF17* in regulating kernel development. Additionally, it is plausible that the other eight transcription factors regulated by miRNA exert crucial roles in the intricate process of kernel development. For instance, the gene *Zm00001d053819*, which is responsible for encoding AUXIN RESPONSE FACTOR 16 (ARF16) among these eight genes, has been identified as a potential candidate gene that regulates agronomic traits related to yield and may have an impact on kernel development [41]. These genes may play an important role in the breeding of high-yielding maize varieties.

### 3.2. DEK219 May Impact Kernel Development by Altering the Efficiency of miRNA Biogenesis

The expression of the majority of miRNAs is repressed in the mutant *dek219* [5], and miRNAs play a critical role in maize kernel development. The involvement of miR169o in maize kernel development has been previously reported, and its overexpression in maize has demonstrated an increase in both kernel size and weight; the repression of miR169o expression results in a reduction in kernel size [42]. Maize miR164e plays a pivotal role in kernel development, as its overexpression in *Arabidopsis* results in impaired seed formation [14]. The overexpression of rice miR397 exhibits a propensity to augment both kernel size and panicle branching, thereby leading to a substantial enhancement in yield [43]. Furthermore, the inhibition of rice miR1432 significantly increased the kernel filling rate and kernel weight [44]. These studies indicated that miRNAs play a crucial role in kernel development. Natural variations in *DEK219* were significantly associated with kernel length [5], which further indicates the important role of *DEK219* in regulating the kernel length. Among these natural variations, SNP4473 causes amino acid changes. This variation results in the substitution of the glycine residue at position 40 in the DEK219 PAZ domain with aspartic acid (Figure S1A). The PAZ domain, named after the proteins Piwi, Argonaut, and Zwille, interacts with one end of double-stranded RNA and plays a crucial role in miRNA biogenesis [45,46]. We employed AlphaFold2 [47] to predict the three-dimensional structure of the PAZ domain when SNP4473 is glycine and aspartic acid, respectively. When SNP4473 is glycine, the region spanning from alanine at position 51 to leucine at position 54 in the PAZ domain exhibits irregular curling (Figure S1B). However, in the case in which SNP4473 is substituted with aspartic acid, this region exhibits an alpha helical conformation (Figure S1C). Further analysis using AlphaFold2 was conducted on the interaction between amino acids. When SNP4473 is glycine, a hydrogen bond is established between this site and alanine at position 51 of the PAZ domain, while aspartic acid at position 50 forms a hydrogen bond with lysine at position 52 (Figure S1D). However, when SNP4473 is aspartic acid, this site forms a hydrogen bond not only with alanine at position 51 but also with lysine at position 52. Furthermore, the aspartic acid at position 50 forms not only a hydrogen bond with lysine at position 52 but also an ionic interaction (Figure S1E). The differences in the interactions between these amino acids may be the reason for the structural variations in the three-dimensional structure from alanine at position 51 to leucine at position 54. The variation at this site may affect the binding efficiency between DEK219 and double-stranded RNA, thereby potentially influencing the biogenesis of miRNAs. *DEK219* may affect the kernel length by modulating the efficiency of miRNA biogenesis.

## 4. Materials and Methods

### 4.1. Plant Materials

The plant materials used in this study included maize inbred line W22, B73, and mutant *dek219* (stored within our laboratory), with its wildtype being W22. And an ethyl methane sulfonate (EMS)-mutagenized stop-gained mutant *hsf17* was obtained in 2022 from the Maize EMS-induced Mutant Database (MEMD; http://maizeems.qlnu.edu.cn/, accessed on 10 March 2022) [48]. The mutants in the MEMD were obtained by mutagenesis of B73 as the wildtype. All plant materials were grown at the Chongzhou Modern Agricultural Research and Development Base, Sichuan Agricultural University. The row length was 3 m, and the row width was 0.6 m, with a spacing of 0.3 m between the plants within rows.

### 4.2. Measurement of Kernel Length

We collected mature kernels from the middle region of 10 well-filled ears of W22, B73, *dek219*, and *hsf17*, respectively. The kernel lengths were examined by randomly selecting 10 kernels of each ear. The average value of 10 measurements was taken as the kernel length for each material. Microsoft Excel (2016) software was used to calculate the *p*-value using the paired two-tailed Student’s *t*-test method.

### 4.3. miRNAs and Transcriptome Analysis

The kernels of W22 and *dek219*, with the pericarp removed, were harvested at 15 days after pollination (DAP). Three independent biological replicates were collected, with each replicate sampled from the central region of a separate well-filled ear. The samples were promptly flash-frozen in liquid nitrogen and subsequently stored at −80 °C for subsequent RNA extraction. The total RNA from each sample was extracted using Trizol reagent (Invitrogen, Carlsbad, CA, USA). The RNA samples were subjected to quality control, library construction, and miRNA and transcriptome sequencing by LC-Biotech Co., Ltd., Hangzhou, China. The raw sequence data for the miRNAs and transcriptome in this study can be accessed from the National Center for Biotechnology Information Sequence Read Archive (http://www.ncbi.nlm.nih.gov/sra, accessed on 1 January 2024) under accession number SRP375987.

### 4.4. Correlation Analysis

The correlation between the transcription factors and the kernel length genes *KL1*, *KL9*, and *DE18* was analyzed using SPSS software (v.24.0). A correlation between transcription factors and kernel length regulation is considered when the Pearson’s correlation coefficient is ≤−0.60 [49].

### 4.5. Transient Expression Analysis

The coding sequences of *HSF17* were cloned and inserted into the effector construct PUbi:*β-GLUCURONIDASE* (*GUS*) by replacing the *GUS* reporter gene and driven by the maize ubiquitin promoter (pUbi:*Hsf17*). The promoter of *DE18* was inserted into the reporter construct pPromoter:*LUCIFERASE* (*LUC*). pUbi:*GUS* was used as an internal construct. The reporter construct, the effector construct, and the internal construct were subsequently combined in a molar ratio of 2:2:1 and co-transformed into maize leaf protoplasts. Three biological replicates were performed. The steps for the preparation and transformation of maize leaf protoplasts can be found in a previous study [5]. The protoplasts were cultured in darkness at 28 °C for 12 h, and the activities of GUS and LUC were quantified using a Luminoskan™ Ascent (Thermo, Waltham, MA, USA). Microsoft Excel (2016) software was used to calculate the *p*-value using the paired two-tailed Student’s *t*-test method. The primers utilized for transient expression analysis are listed in Table S4.

### 4.6. Electrophoretic Mobility Shift Assay

The coding sequence of *Hsf17* was cloned into pET32a, and recombinant His-*Hsf17* was purified using the Ni-NTA His Bind purification Kit (Novagen, Darmstadt, Germany) following the manufacturer’s instructions. The oligonucleotide probe was synthesized and labeled with biotin at the 5′ end by Songon (Shanghai, China). Native-PAGE was used for electrophoresis. After electrophoresis, the binding reactions were transferred to a nylon membrane, and then, the transferred DNA was crosslinked to the membrane by UV light. The detection of biotin-labeled DNA was performed using the LightShift Chemiluminescent EMSA kit (Thermo Scientific). The probe sequence is listed in Table S4.

### 4.7. Bioinformatics Analysis

Prediction of the miRNA-target genes was accomplished using the psRNATarget tool (https://www.zhaolab.org/psRNATarget/, accessed on 1 March 2022) [50], expectation ≤ 4. The analysis of the protein three-dimensional structure and the interactions between amino acids was accomplished using AlphaFold2 [47].

## 5. Conclusions

Initially, we identified a gene *DEK219* that exhibits association with the kernel length of maize. Further identification revealed that there were nine transcription factors regulated by *DEK219* and potentially affecting the kernel length. The investigation revealed that *HSF17*, one of these transcription factors, functions as a negative regulator of kernel length. The present study has provided novel genetic resources to optimize the breeding of high-yielding maize varieties.

## Data Availability

The data are contained within the article and Supplementary Materials.

## Supplementary Materials

The following supporting information can be downloaded at https://www.mdpi.com/article/10.3390/plants13121592/s1: Table S1: The expression levels of 155 transcription factors were significantly upregulated in *dek219*; Table S2: The results of miRNA-targeted transcription factor prediction; Table S3: The correlation between the 27 transcription factors and kernel length genes *KL1*, *KL9*, and *DE18*; Table S4: Primers and probe used in this study; Figure S1: The natural variation in *DEK219* may affect the three-dimensional structure of its protein. (A) Schematic diagram of the *DEK219* gene and conserved domains. Empty boxes represent the 5′ and 3′ untranslated regions, while other boxes represent coding regions, and lines represent introns. SNP, single-nucleotide polymorphism; AA, amino acids; DEXDc, DEAD-like helicases superfamily; HELICc, helicase superfamily c-terminal domain; PAZ, Piwi Argonaut and Zwille; RNase III, Ribonuclease III family; dsRBD, double-stranded RNA binding domains. (B) When SNP4473 is glycine, the region from the 51st alanine to the 54th leucine in the PAZ domain exhibits irregular curling. This area indicated by the arrow. (C) When SNP4473 is aspartic acid, the region from the 51st alanine to the 54th leucine in the PAZ domain exhibits alpha helical conformation. This area indicated by the arrow. (D) When SNP4473 is glycine, the interaction among the neighboring amino acids at this site. The arrows indicate the interaction between amino acids. (E) When SNP4473 is aspartic acid, the interaction among the neighboring amino acids at this site. The arrows indicate the interaction between amino acids.
