# Supplementary material for: DEK219 and HSF17 Collaboratively Regulate the Kernel Length in Maize
Source: Plants (Basel). 2024 Jun 7;13(12):1592. doi: 10.3390/plants13121592 (PMC11207566; doi:10.3390/plants13121592)
Supplement: Supplementary file 1 [file plants-13-01592-s001.zip › Supplementary Materials/Supplementary Figure S1.pdf]

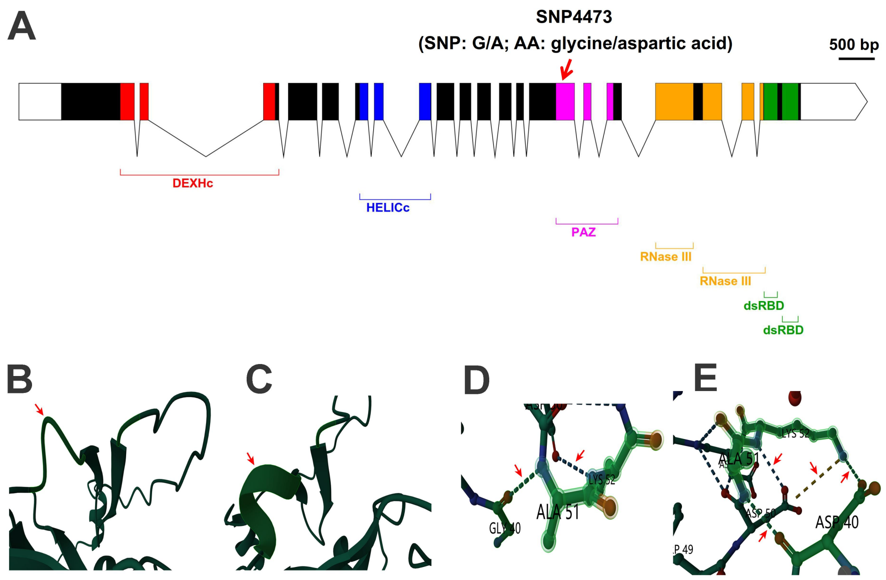

Figure S1 The natural variation in *DEK219* may affect the three-dimensional structure of its protein. (**A**) Schematic diagram of the *DEK219* gene and conserved domains. Empty boxes represent the 5' and 3' untranslated regions, while other boxes represent coding regions, and lines represent introns. SNP, single-nucleotide polymorphism; AA, amino acids; DEXDc, DEAD-like helicases superfamily; HELICc, helicase superfamily c-terminal domain; PAZ, Piwi Argonaut and Zwille; RNase III, Ribonuclease III family; dsRBD, double-stranded RNA binding domains. (**B**) When SNP4473 is glycine, the region from the 51st alanine to the 54th leucine in the PAZ domain exhibits irregular curling. This area indicated by the arrow. (**C**) When SNP4473 is aspartic acid, the region from the 51st alanine to the 54th leucine in the PAZ domain exhibits alpha helical conformation. This area indicated by the arrow. (**D**) When SNP4473 is glycine, the interaction among the neighboring amino acids at this site. The arrows indicate the interaction between amino acids. (**E**) When SNP4473 is aspartic acid, the interaction among the neighboring amino acids at this site. The arrows indicate the interaction between amino acids.
